# Estimated glucose disposal rate and severe abdominal aortic calcification: evidence from a nationally representative study with external validation

**DOI:** 10.3389/fnut.2026.1790028

**Published:** 2026-06-19

**Authors:** Wenjun Liu, Jijun Wu, Jiawei Peng, Yiduo Bai, Hao Liu, Jianmin Liang

**Affiliations:** 1 Division of Vascular and Interventional Radiology, Department of General Surgery, Nanfang Hospital, Southern Medical University, Guangzhou, China; 2 Department of Vascular Surgery, Guangdong Provincial Key Laboratory of Major Obstetric Diseases, Guangdong Provincial Clinical Research Center for Obstetrics and Gynecology, The Third Affiliated Hospital, Guangzhou Medical University, Guangzhou, China; 3 Department of Interventional Radiology, Zhongshan Torch Development Zone People's Hospital, Zhongshan, China; 4 The First Department of General Surgery, The First Affiliated Hospital of Guangdong Pharmaceutical University, Guangzhou, China

**Keywords:** abdominal aortic calcification, estimated glucose disposal rate, external validation, insulin resistance, NHANES, severe abdominal aortic calcification

## Abstract

**Background:**

Estimated glucose disposal rate (eGDR) is a validated surrogate marker of insulin resistance and has been associated with cardiometabolic risk. However, its relationship with abdominal aortic calcification (AAC) remains incompletely understood, and evidence from independent populations is limited.

**Methods:**

We analyzed data from 2,994 adults aged ≥20 years in the National Health and Nutrition Examination Survey (NHANES) 2013–2014. An independent external validation cohort including 745 participants was used to confirm the findings. Severe AAC was defined using the Kauppila scoring system. Multivariable logistic regression models were applied to evaluate the association between eGDR and severe AAC. Dose–response relationships were assessed using restricted cubic spline models, and subgroup analyses were performed to explore potential effect modification.

**Results:**

Lower eGDR levels were significantly associated with higher odds of severe AAC in both the NHANES cohort (fully adjusted OR per 1-unit increase: 0.82; 95% CI: 0.75–0.90) and the validation cohort (OR: 0.79; 95% CI: 0.69–0.91). Participants in the highest eGDR tertile had substantially lower odds of severe AAC compared with those in the lowest tertile in both cohorts. Restricted cubic spline analyses demonstrated an approximately linear inverse association between eGDR and severe AAC, with no evidence of nonlinearity. Similar findings were observed in the external validation cohort. Subgroup analyses showed consistent associations across most population strata.

**Conclusion:**

Lower eGDR is independently and linearly associated with a higher prevalence of severe AAC. These findings, validated in an independent cohort, support the potential utility of eGDR as a simple and accessible marker for identifying individuals at increased risk of vascular calcification.

## Introduction

Vascular calcification, a hallmark of advanced atherosclerosis, has emerged as a critical pathological process linking metabolic disorders to adverse cardiovascular outcomes. It reflects cumulative vascular injury and is strongly associated with increased cardiovascular morbidity and mortality. AAC, a specific form of vascular calcification, is clinically important and is widely recognized as a marker of systemic atherosclerotic burden. Moreover, AAC has been independently associated with an increased risk of cardiovascular events and all-cause mortality (1, 2).

Compared with coronary artery calcification, AAC can be detected through easy available imaging techniques, such as lateral dual-energy X-ray absorptiometry, and often precedes clinically overt cardiovascular disease (3, 4). Importantly, AAC has been recognized as a robust marker of subclinical atherosclerosis and arterial stiffness, providing complementary prognostic information beyond traditional cardiovascular risk factors. Accumulating evidence suggests that AAC is not merely a passive degenerative process associated with aging, but rather a regulated phenomenon influenced by complex interactions among metabolic, inflammatory, and hemodynamic factors (5). With population aging and the growing prevalence of metabolic diseases, early identification of individuals at high risk for AAC has become an urgent public health priority (6).

Insulin resistance is a key pathophysiological mechanism underlying the development of atherosclerosis and vascular calcification through multiple interrelated pathways, including chronic low-grade inflammation, endothelial dysfunction, oxidative stress, and metabolic dysregulation (7, 8). Experimental studies have demonstrated that insulin resistance can induce osteogenic differentiation of vascular smooth muscle cells, increase inflammatory cytokine production, and promote calcium deposition within the arterial wall. Epidemiological and clinical studies have also consistently linked insulin resistance to vascular calcification. Specifically, insulin resistance has been shown to be directly associated with increased AAC burden in population-based studies. Individuals with higher levels of insulin resistance tend to exhibit higher AAC scores (9, 10). Several indices have been used to assess insulin resistance, including fasting insulin, the homeostasis model assessment of insulin resistance (HOMA-IR), and glycemic markers such as fasting plasma glucose and glycated hemoglobin (HbA1c) (11). Although these indicators have contributed to our understanding of metabolic – vascular interactions, their clinical applicability remains limited. Insulin measurements are not routinely available in large-scale population studies, and single glycemic measures may not adequately capture the complex metabolic disturbances associated with insulin resistance (12).

Estimated glucose disposal rate (eGDR) has emerged as a practical and validated surrogate marker of insulin resistance that incorporates waist circumference, hypertension status, and HbA1c, which are readily available in routine clinical practice (13). Originally developed in individuals with diabetes, eGDR has subsequently been validated in broader populations and shown to outperform traditional glycemic indices in predicting cardiometabolic risk (14). Lower eGDR values have been associated with an increased risk of peripheral vascular disease and coronary artery disease, suggesting that it may reflect systemic atherosclerotic burden (15, 16). The eGDR is clinically more sensible and applicable and offers greater feasibility, stability, and clinical interpretability compared with conventional indices of insulin resistance.

Despite its widespread use as a surrogate marker of insulin resistance, evidence linking estimated glucose disposal rate (eGDR) to subclinical vascular structural changes remains limited. In particular, the relationship between eGDR and AAC, assessed using standardized imaging techniques, has not been fully elucidated. In addition, most existing studies have relied on single cohorts, which may limit the generalizability and reproducibility of findings. A recent nationwide study based on NHANES 2013–2014 data reported a significant inverse association between eGDR and severe AAC (17). Notably, that study identified a nonlinear dose–response relationship with a threshold effect. However, it relied solely on a single cross-sectional cohort, which may limit the generalizability and reproducibility of the findings. In addition, the lack of detailed reporting on participant selection strategies may introduce uncertainty regarding potential selection bias. Therefore, several important questions remain unresolved. Importantly, whether this association can be consistently replicated in independent populations and whether the dose–response relationship remains stable across different populations remain unclear.

To address these gaps, the present study aimed to investigate the association between eGDR and severe AAC in a nationally representative population using NHANES 2013–2014 data, and to further validate these findings in an independent external cohort. By integrating population-based analysis with external validation, this study not only confirms previous findings but also extends the existing evidence by demonstrating a consistent and approximately linear association across independent populations, thereby improving the robustness and generalizability of the observed relationship.

## Methods

### Study design and population

**Figure 1:**
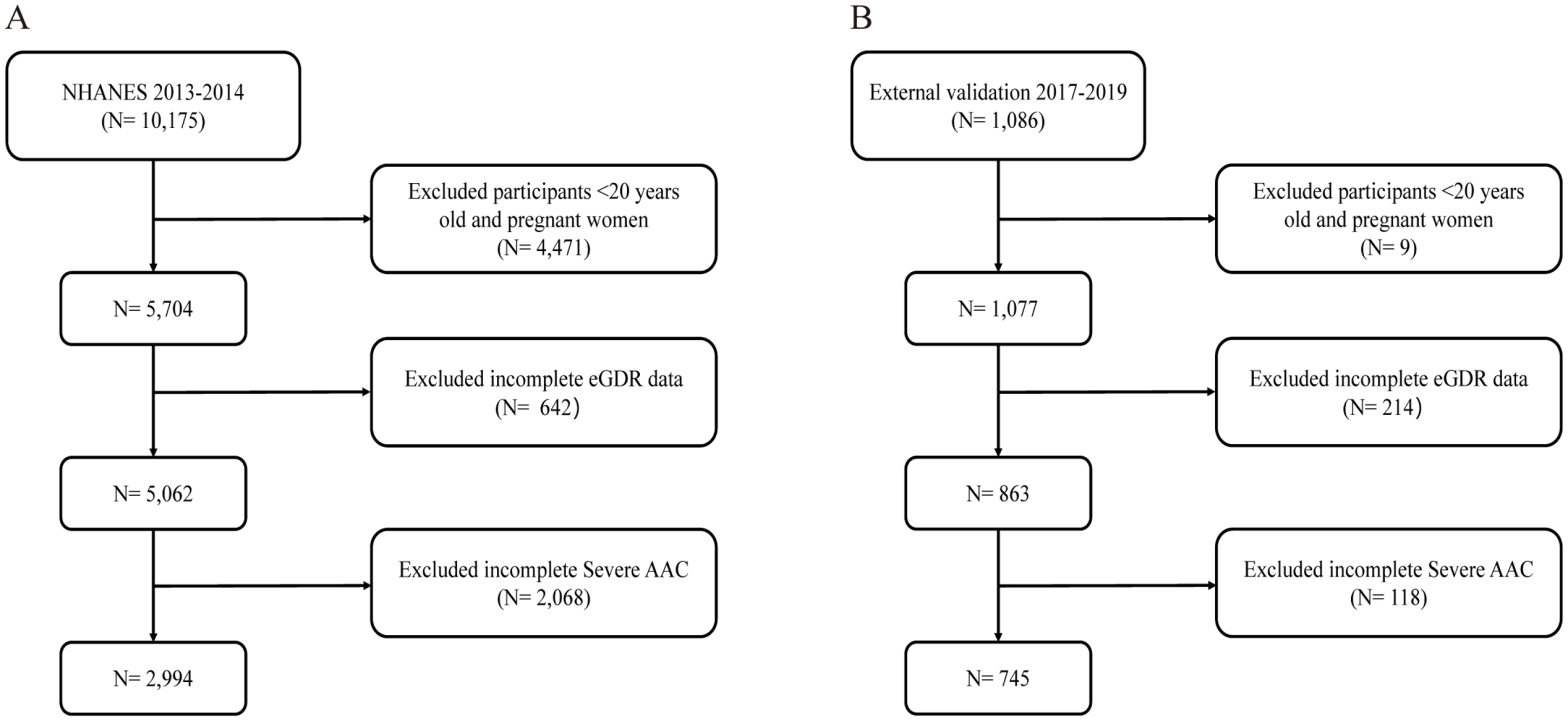
**(A)** Flow diagram of participant selection in the NHANES 2013–2014 cohort. **(B)** Flow diagram of participant selection in the external validation cohort. Abbreviations: AAC, abdominal aortic calcification; CCI, Charlson Comorbidity Index; eGDR, estimated glucose disposal rate.

For the purpose of the cross-sectional study, the authors had used publicly available data of the National Health and Nutrition Examination Survey (NHANES) 2013–2014. The NHANES survey is a nation-wide survey designed by the Centres for Disease Control and Prevention (CDC) to determine the health and nutritional status of the non-institutionalized U. S. (18). The study utilized information from adult volunteers, aged 20 years and older, who had recorded information of estimated glucose disposal rate, or eGDR, and AAC. We excluded participants who were: (1) pregnant or younger than 20 years old (*n* = 4,471), as this study focused on adult populations and pregnancy is associated with physiological changes that may substantially alter metabolic parameters, including glycemic status and blood pressure, which are components of eGDR; (2) missing data on eGDR (*n* = 642); (3) missing AAC data (n = 2,068). Ultimately, 2,994 eligible subjects were enrolled in the final analysis (Figure 1A). Supplementary Table S1 presents definitions of variables used in this study.

To validate our findings, we conducted an independent validation cohort analysis using data from the First Affiliated Hospital of Guangdong Pharmaceutical University collected between 2017 and 2019. This retrospective cohort included adults aged ≥20 years who underwent routine health examinations with available data for eGDR calculation and AAC assessment. We initially identified 1,086 individuals with available lumbar spine DXA scans. Participants were excluded if they were (1) aged <20 years or pregnant (*n* = 9), for the same reasons as described above, including the focus on adult populations and the potential influence of pregnancy-related physiological changes on metabolic indicators, (2) missing data required to calculate eGDR (*n* = 214), or (3) missing AAC assessment or having DXA images of insufficient quality for Kauppila scoring (*n* = 118). In all, 745 participants were included in the final validation analysis who met the inclusion criteria (Figure 1B).

The NHANES and Validation Cohort were approved by the National Center for Health Statistics Research Ethics Review Board and the Ethics Review Committees of the First Affiliated Hospital of Guangdong Pharmaceutical University, respectively. Each participant in both cohorts provided informed consent. The reporting of this study conformed to STROBE (Strengthening the Reporting of Observational Studies in Epidemiology) guidelines (19).

### Assessment of estimated glucose disposal rate (eGDR)

eGDR was calculated using a validated formula: eGDR = 21.158 – (0.09 × waist circumference [cm]) – (3.407 × hypertension [yes = 1, no = 0]) – (0.551 × HbA1c [%]). In both cohorts, waist circumference was measured by trained technicians. Hypertension was defined based on physician diagnosis or antihypertensive medication use. HbA1c was determined from whole blood samples using standardized laboratory methods and quantified by high-performance liquid chromatography (11).

### Definitions of AAC

The AAC was assessed using lateral dual-energy X-ray absorptiometry (DXA) scans of the lumbar spine (L1–L4) following the Kauppila scoring system. This semi-quantitative method assigns scores from 0 to 24 based on the presence and severity of calcific deposits along the anterior and posterior aortic walls adjacent to each vertebra. Severe AAC was defined as a Kauppila score >6 in line with previous literature (20, 21).

### Assessment of covariates

Covariates were selected based on prior literature and clinical relevance. Sociodemographic variables included age, sex, race/ethnicity (in NHANES only), education level, marital status, and poverty-income ratio. Lifestyle factors included smoking status (never, former, current), alcohol consumption (never, former, current), and obesity (body mass index ≥30 kg/m^2^). The Charlson Comorbidity Index (CCI) was calculated to reflect overall comorbidity burden (Supplementary Table S2). All covariates were harmonized between the two cohorts to ensure comparability.

### Statistical analysis

Mean values and values of the standard deviation (SD) were reported for continuous variables. Counts and proportions were used to characterize variables. We carried out analysis of variance continuous variables and chi square test categorical variables to show baseline characteristics in the three groups of eGDR tertiles. To deal with the missing data of covariates, multiple imputations with chained equations were used (22). For each dataset, we derived independent effect estimates and pooled them using Rubin’s rules. The R package mice was used to carry out the multiple imputations.

Three models were fitted for the Cox regression. Model 1: unadjusted; Model 2: adjusted for age, sex, and race/ethnicity (only in NHANES); Model 3: further adjusted for education, marital status, PIR, smoking, alcohol use, obesity, and CCI. All analyzes were conducted in R version 4.4.2 and Stata 17.0. A two-tailed *p*-value <0.05 was considered statistically significant (23). Restricted cubic spline (RCS) regression was used to assess the possible linear relationship between eGDR and AAC. We performed subgroup analyses and tested interaction significance using likelihood ratio tests.

## Results

### Baseline characteristics

A total of 2,994 participants from NHANES (female: 52.0%, mean age: 57.4 years) and 745 from the validation cohort (female: 52.6%, mean age: 59.8 years) were included according to the inclusion and exclusion criteria. The baseline characteristics of these participants stratified by severe AAC status are shown in Table 1 (NHANES) and Table 2 (validation cohort). In both cohorts, participants with severe AAC were older, with a significantly higher proportion aged over 65 years (NHANES: 73.1% vs. 22.3%; validation cohort: 56.4% vs. 34.5%; both *p* < 0.001). Those with severe AAC also had lower educational attainment, with a higher proportion having education below high school level (NHANES: 23.0% vs. 15.0%; validation cohort: 54.5% vs. 36.3%; both *p* < 0.05).

**Table 1:** Baseline characteristics of all participants were stratified by Severe AAC in NHANES cohort.

Characteristic	Overall, *N* = 2,994 (100%)	Non-Severe AAC *N* = 2,726 (92.2%)	Severe AAC *N* = 268 (7.8%)	*p*-value
Age (%)				<0.001
20–65	2,079 (74%)	2,010 (78%)	69 (27%)	
>65	915 (26%)	716 (22%)	199 (73%)	
Sex (%)				0.50
Female	1,541 (52%)	1,403 (51%)	138 (55%)	
Male	1,453 (48%)	1,323 (49%)	130 (45%)	
Race (%)				0.013
Non-Hispanic White	1,325 (71%)	1,151 (71%)	174 (80%)	
Other	697 (12%)	660 (12%)	37 (9.4%)	
Non-Hispanic Black	577 (9.9%)	543 (10%)	34 (6.2%)	
Mexican American	395 (6.9%)	372 (7.2%)	23 (4.1%)	
Married/live with partner (%)				<0.001
No	1,067 (31%)	934 (29%)	133 (50%)	
Yes	1,926 (69%)	1,791 (71%)	135 (50%)	
Education level (%)				0.023
Below high school	685 (15%)	614 (15%)	71 (23%)	
High School or above	2,307 (85%)	2,110 (85%)	197 (77%)	
PIR (%)				0.13
Poor	826 (19%)	748 (19%)	78 (23%)	
Not Poor	1,930 (81%)	1,757 (81%)	173 (77%)	
Obesity (%)				0.002
No	1,928 (64%)	1,725 (63%)	203 (76%)	
Yes	1,059 (36%)	994 (37%)	65 (24%)	
Smoking (%)				0.001
Never	1,610 (54%)	1,507 (56%)	103 (37%)	
Former	831 (28%)	721 (27%)	110 (43%)	
Current	552 (17%)	497 (17%)	55 (20%)	
Drinking (%)				<0.001
Never	417 (11%)	388 (11%)	29 (10%)	
Former	585 (17%)	502 (16%)	83 (30%)	
Mild	1,058 (41%)	959 (41%)	99 (42%)	
Moderate	393 (17%)	372 (17%)	21 (6.7%)	
Heavy	371 (14%)	344 (15%)	27 (11%)	
CCI [mean (SD)]	1.33 (1.65)	1.24 (1.60)	2.39 (1.85)	<0.001
eGDR [mean (SD)]	7.29 (2.53)	7.38 (2.55)	6.20 (1.99)	<0.001
eGDR, Tertile (%)				<0.001
T1	1,063 (33%)	935 (32%)	128 (46%)	
T2	1,015 (33%)	907 (33%)	108 (40%)	
T3	916 (33%)	884 (35%)	32 (13%)	

Mean (SD) for continuous variables: the *p*-value was calculated by the weighted Students *T*-test. Percentages (95%CI) for categorical variables: the *p*-value was calculated by the weighted chi-square test.

AAC, abdominal aortic calcification; CCI, Charlson Comorbidity Index; eGDR, estimated glucose disposal rate; PIR, poverty income ratio.

**Table 2:** Baseline characteristics of individuals classified by tertiles of eGDR in the validation cohort.

Variables	Total (*n* = 745)	0 (*n* = 644)	1 (*n* = 101)	Statistic	*P*
CCI, Mean ± SD	1.37 ± 1.62	1.25 ± 1.59	2.09 ± 1.66	*t* = −4.88	**<0.001**
eGDR, Mean ± SD	6.88 ± 2.45	7.02 ± 2.48	5.97 ± 2.04	*t* = 4.65	**<0.001**
Sex, *n* (%)				*χ*^2^ = 0.21	0.646
Female	392 (52.62)	341 (52.95)	51 (50.50)		
Male	353 (47.38)	303 (47.05)	50 (49.50)		
Marital status, *n* (%)				*χ*^2^ = 9.78	**0.002**
No	300 (40.27)	245 (38.04)	55 (54.46)		
Yes	445 (59.73)	399 (61.96)	46 (45.54)		
Education, *n* (%)				*χ*^2^ = 12.07	**<0.001**
Below high school	289 (38.79)	234 (36.34)	55 (54.46)		
High School or above	456 (61.21)	410 (63.66)	46 (45.54)		
PIR, *n* (%)				*χ*^2^ = 0.05	0.819
Not Poor	509 (68.32)	439 (68.17)	70 (69.31)		
Poor	236 (31.68)	205 (31.83)	31 (30.69)		
Obesity, *n* (%)				*χ*^2^ = 3.96	**0.047**
No	488 (65.50)	413 (64.13)	75 (74.26)		
Yes	257 (34.50)	231 (35.87)	26 (25.74)		
Smoking, *n* (%)				*χ*^2^ = 15.94	**<0.001**
Current	147 (19.73)	128 (19.88)	19 (18.81)		
Former	245 (32.89)	195 (30.28)	50 (49.50)		
Never	353 (47.38)	321 (49.84)	32 (31.68)		
Drinking, *n* (%)				*χ*^2^ = 9.80	**0.044**
Former	175 (23.49)	142 (22.05)	33 (32.67)		
Heavy	92 (12.35)	85 (13.20)	7 (6.93)		
Mild	279 (37.45)	240 (37.27)	39 (38.61)		
Moderate	101 (13.56)	93 (14.44)	8 (7.92)		
Never	98 (13.15)	84 (13.04)	14 (13.86)		
Age custom, *n* (%)				*χ*^2^ = 17.98	**<0.001**
1	466 (62.55)	422 (65.53)	44 (43.56)		
2	279 (37.45)	222 (34.47)	57 (56.44)		

Bold values indicate statistical significance (*p* < 0.05).

Participants with severe AAC exhibited distinct metabolic and clinical profiles. They had significantly lower eGDR levels in both cohorts (NHANES: 6.20 vs. 7.38; validation cohort: 5.97 vs. 7.02; both *p* < 0.001) and higher comorbidity burden as reflected by Charlson Comorbidity Index scores (NHANES: 2.39 vs. 1.24; validation cohort: 2.09 vs. 1.25; both *p* < 0.001). Lifestyle factors also differed, with the severe AAC groups having higher proportions of former smokers and former drinkers. In both cohorts, the highest eGDR tertile was underrepresented among participants with severe AAC (NHANES: 13.0% vs. 35.0%; validation cohort: 13.0% vs. 35.0%; both *p* < 0.001), while the lowest tertile was overrepresented (NHANES: 46.0% vs. 32.0%; validation cohort: 46.0% vs. 32.0%; both p < 0.001). Overall, these findings suggest that individuals with lower eGDR levels tend to have a less favorable cardiometabolic profile and a higher burden of severe AAC.

### Association between eGDR and severe AAC

In the NHANES cohort, each 1-unit increase in eGDR was associated with a 17% reduction in the odds of severe AAC in the unadjusted model (OR = 0.83; 95% CI: 0.80–0.87; *p* < 0.001). When a full adjustment was done for demographic characteristics, socioeconomic factors, lifestyle variables, and the burden of comorbidity (model 3), the association remained significant, such that each 1-unit eGDR increase was associated with an 18% decreased odds of severe AAC (OR = 0.82; 95% CI: 0.75–0.90; *p* < 0.001) (Table 3). A clear dose–response relationship emerged upon examination by eGDR tertiles. Subjects placed in highest tertile eGDR (T3) had 74% lower odds of severe AAC compared to the lowest tertile eGDR (T1) in the fully adjusted model (OR = 0.26; 95% CI: 0.15–0.46; *p* < 0.001). A similar trend was observed for the middle tertile (T2), although the association did not reach statistical significance. The *P* for trend across tertiles was consistently significant (0.001 in crude model, 0.010 in fully adjusted model).

**Table 3:** Association between eGDR and Severe AAC in NHANES cohort.

Characteristics	Model 1 [OR (95% CI)]	*p*-value	Model 2 [OR (95% CI)]	*p*-value	Model 3 [OR (95% CI)]	*p*-value
Continuous	0.83(0.80,0.87)	<0.001	0.90(0.84, 0.96)	0.010	0.82(0.75,0.90)	<0.001
Tertile						
T1	1 (ref.)		1 (ref.)		1 (ref.)	
T2	0.86(0.67,1.11)	0.070	0.99(0.72, 1.38)	0.980	0.64(0.43,0.96)	0.030
T3	0.26(0.15,0.46)	<0.001	0.48(0.23, 0.99)	0.047	0.37(0.18,0.74)	0.010
*P* for trend	<0.001		0.046		0.010	

Model 1: no covariates were adjusted. Model 2: age, sex, and race were adjusted. Model 3: age, sex, education level, marital status, PIR, race, obesity, smoking, drinking, and CCI were adjusted.

AAC, abdominal aortic calcification; CCI, Charlson Comorbidity Index; eGDR, estimated glucose disposal rate; PIR, poverty income ratio; OR, odds ratio; CI, confidence interval.

In the independent validation cohort, there was a 16% reduction in odds of severe AAC with each 1-unit increase in eGDR in the unadjusted model (OR = 0.84; 95% CI: 0.77–0.91; *p* < 0.001). Following complete adjustment for the same covariates as the NHANES data (Model 3), the association mainly held; with each 1 unit increase in eGDR, severe AAC odds decreased by 21% (OR = 0.79; 95% CI:0.69–0.91; *p* < 0.001). When categorized into tertiles, a clear dose–response relationship was observed. Compared to participants in the lowest eGDR tertile (T1), participants in the highest eGDR tertile (T3) exhibited 72% lower odds of severe AAC in the fully adjusted model (OR = 0.28; 95% CI: 0.13–0.61; *p* < 0.001). The middle tertile (T2) showed a non-significant reduced odds trend. The *P* for trend across tertiles was highly significant (0.003 in fully adjusted model) (Table 4). Overall, these results indicate a consistent inverse association between eGDR and the risk of severe AAC across both cohorts.

**Table 4:** Association between eGDR and Severe AAC in the validation cohort.

Characteristics	Model 1 OR (95%CI)	*p*-value	Model 2 OR (95%CI)	*p*-value	Model 3 OR (95%CI)	*p*-value
Continuous	0.84 (0.77 ~ 0.91)	<0.001	0.86 (0.78 ~ 0.95)	0.003	0.79 (0.69 ~ 0.91)	<0.001
Tertile						
T1	1.00 (Reference)		1.00 (Reference)		1.00 (Reference)	
T2	0.81 (0.51 ~ 1.30)	0.391	0.77 (0.46 ~ 1.27)	0.302	0.63 (0.34 ~ 1.16)	0.139
T3	0.33 (0.18 ~ 0.60)	**<0.001**	0.40 (0.21 ~ 0.76)	**0.005**	0.28 (0.13 ~ 0.61)	**<0.001**
*P* for trend	<0.001		0.005		0.003	

OR, odds ratio; CI, confidence interval.

Model 1: Crude; Model 2: Adjust: Sex, age; Model 3: Adjust: Sex, age, Marital status, Education, PIR, Obesity, Smoking, Drinking, CCI. Bold values indicate statistical significance (*p* < 0.05).

### Dose–response relationship between eGDR and severe AAC

**Figure 2:**
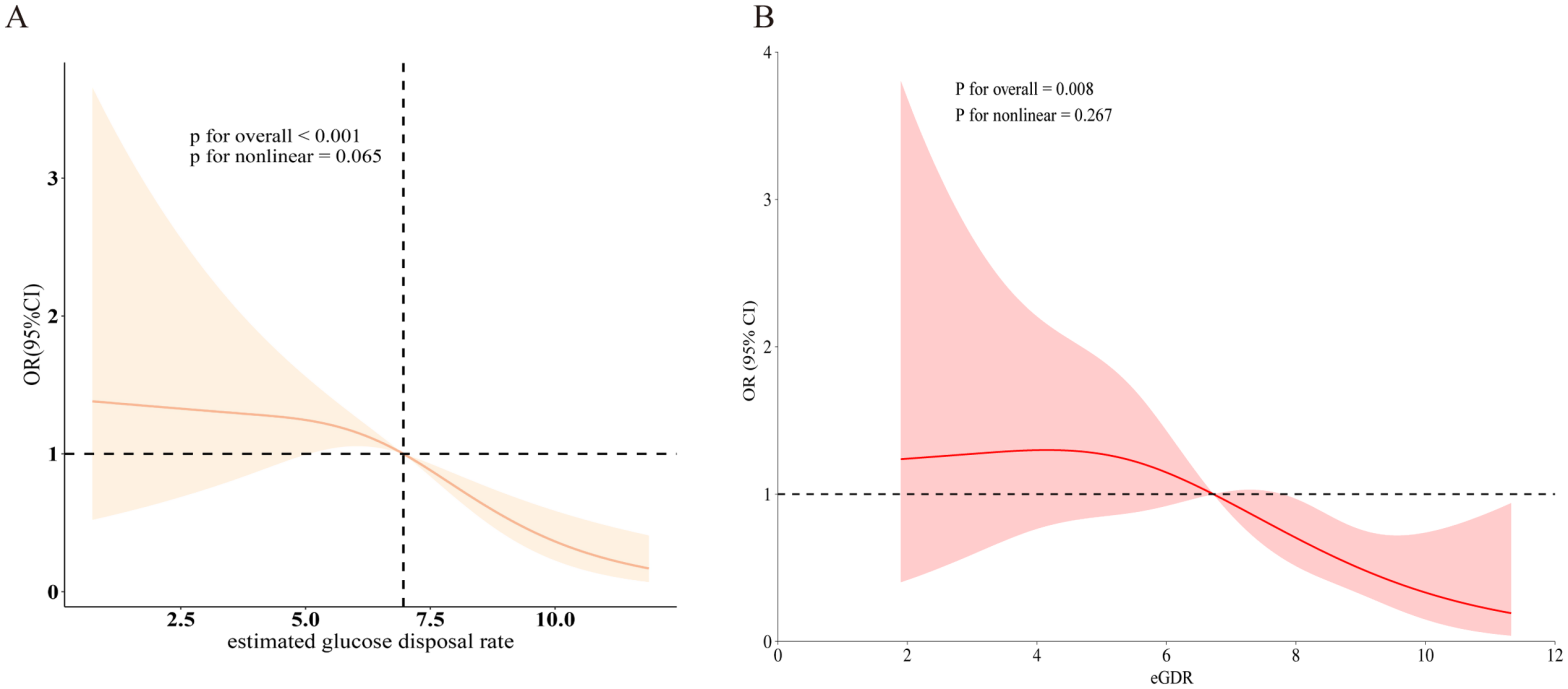
**(A)** Restricted cubic spline analysis showing the dose–response relationship between eGDR and severe AAC in the NHANES cohort. **(B)** Restricted cubic spline analysis showing the dose–response relationship between eGDR and severe AAC in the external validation cohort. OR (solid lines) and 95% confidence intervals (shaded areas) were adjusted for age, sex, education level, marital status, PIR, race (NHANES only), obesity, smoking, drinking, and CCI.

In the validation cohort, similar dose–response patterns were observed (Figure 2B). The association was significant overall (*P* for overall = 0.008), with no evidence of nonlinearity (*P* for nonlinear = 0.267). Restricted cubic spline analysis also demonstrated an approximately linear inverse association between eGDR and severe AAC, with no evidence of nonlinearity (Figure 2A). Similar linear trends were observed in the external validation cohort, supporting the consistency of the findings. The dose–response curve closely resembled that observed in the NHANES cohort, with the odds of severe AAC decreasing progressively as eGDR increased. These findings support a stable and approximately linear inverse relationship between eGDR and severe AAC across different populations.

### Subgroup analyzes

**Figure 3:**
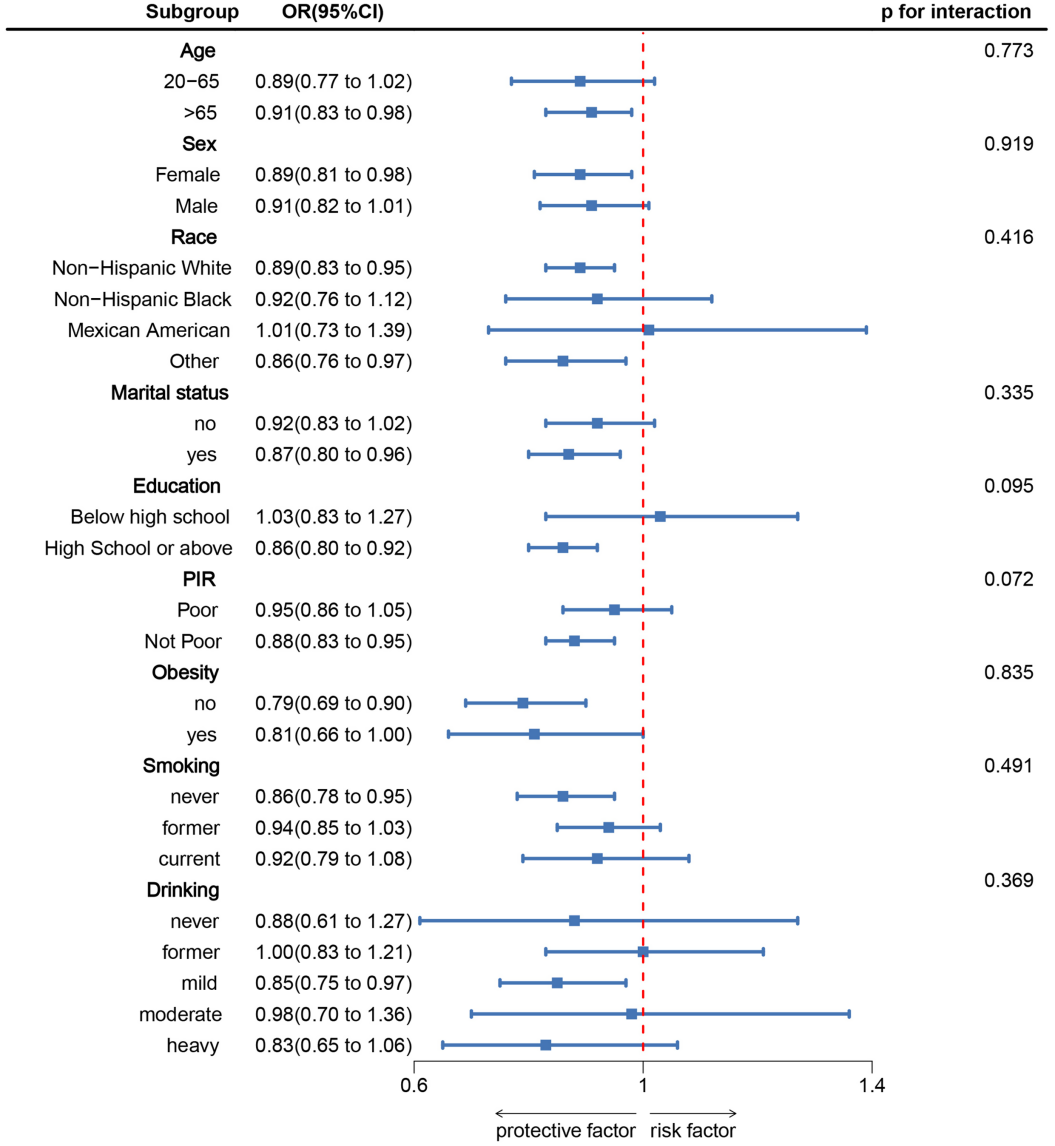
Subgroup analysis between eGDR and Severe AAC in NHANES cohort. ORs were calculated per 1-unit increase in eGDR. Analyzes were adjusted for age, sex, education level, marital status, PIR, race, obesity, smoking, drinking, and CCI.

**Figure 4:**
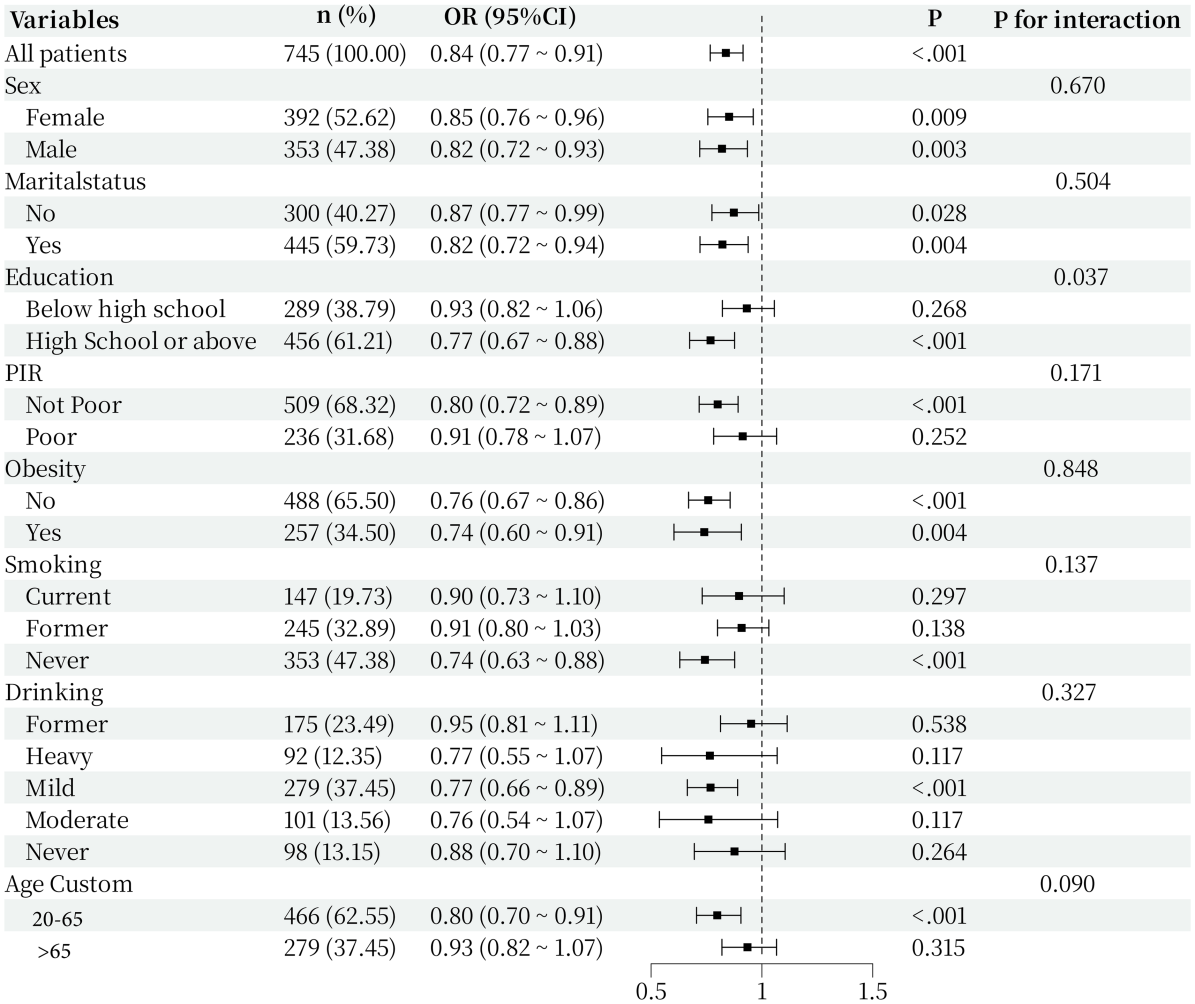
Subgroup analysis between eGDR and Severe AAC in the validation cohort. ORs were calculated per 1-unit increase in eGDR. Analyzes were adjusted for age, sex, education level, marital status, PIR, race, obesity, smoking, drinking, and CCI.

Subgroup analyses revealed consistent associations between higher eGDR and reduced severe AAC odds across both cohorts, with notable effect modifications (Figure 3, NHANES; Figure 4, validation cohort). The interaction statistics indicate that in the NHANES analysis (OR for >65 years = 0.91; 20–65 years = 0.89; P-interaction = 0.773), protective associations were stronger in older adults, whereas in the validation cohort, it was the opposite (OR for 20–65 years = 0.80; >65 years = 0.93; *P*-interaction = 0.037). Similar patterns were observed across education levels. There were stronger effects for the higher-educated individuals in both cohorts. For the validation cohort, we see high school+ have OR = 0.77 and below high school OR = 0.93. *P*-interaction is 0.171. The associations were not meaningfully modified by obesity status in either cohort. Evidence showed consistent protective effects across all body mass index (BMI) categories (validation cohort: non-obese: odds ratio (OR) = 0.76, obese: OR = 0.74, *P*-interaction = 0.848). Never-smokers showed strongest protection effect in validation cohort (OR = 0.74; *p* < 0.001). Patterns of alcohol consumption showed the mildest drinkers having the strongest association (OR = 0.77; *p* < 0.001). Although the strength of the associations varied across subgroups, the overall direction remained consistent, supporting the robustness of the observed relationship.

## Discussion

Using a representative sample of U. S. adults and an independent external validation cohort, this study assessed the association between estimated glucose disposal rate (eGDR) and severe AAC. We found that lower eGDR levels were consistently linked with a greater prevalence of severe AAC in both cohorts, independent of demographic characteristics, socioeconomic status, lifestyle factors, and comorbidity burden. Furthermore, restricted cubic spline analyses showed that eGDR and severe AAC has an inverse-dose relationship which is approximately linear. The associations remained stable across various sensitivity checks and subgroup assessments and were confirmed in the outside validation group. When assessed together, our results suggest that eGDR is a clinically accessible insulin resistance biomarker that is inversely related to severe AAC. These findings support the potential clinical utility of eGDR for identifying individuals at increased risk for subclinical vascular disease.

To better contextualize our findings, we compared our results with those of previous studies. Our findings are generally consistent with a previous NHANES-based study that demonstrated an inverse association between eGDR and severe AAC risk (1). However, several important differences substantially extend the existing literature. First, unlike the previous study, which relied solely on NHANES data, our study incorporated an independent external validation cohort. The consistency of findings across two distinct populations strengthens the robustness, reproducibility, and generalizability of the observed association. Second, while the prior study reported a nonlinear relationship with a threshold effect, we observed an approximately linear inverse association without a clear threshold. The difference in dose–response patterns between studies is noteworthy and warrants further consideration. Several factors may contribute to this discrepancy. Differences in participant selection strategies and sample characteristics may influence the distribution of eGDR and the observed shape of the association. In particular, variations in exclusion criteria and data completeness may lead to differences in population composition and underlying risk profiles. In addition, differences in covariate adjustment and statistical modeling approaches, including the number and placement of knots in restricted cubic spline analyses, may affect the estimation of nonlinear trends and contribute to different curve shapes across studies. Importantly, the approximately linear association in our study was consistently observed not only in the NHANES cohort but also in the independent external validation cohort, suggesting that this relationship may be more stable and generalizable across populations than previously reported. This consistency across two independent cohorts further supports the robustness of the observed linear relationship. Third, our study provided a more transparent and clearly defined participant selection process with explicit inclusion and exclusion criteria. This methodological clarity enhances reproducibility and reduces the potential for selection bias, thereby strengthening the internal validity of our findings. Taken together, these differences indicate that our study not only confirms previous observations but also extends them by improving methodological transparency, validating the findings in an independent population, and providing new insights into the dose response relationship.

From a biological perspective, several mechanisms may explain the observed association between lower eGDR and an increased risk of severe AAC. As a validated surrogate marker of insulin resistance, lower eGDR reflects impaired insulin sensitivity, which plays a central role in the development of vascular calcification (24, 25). At the vascular level, insulin resistance may promote calcification through multiple interrelated pathways. These include chronic low-grade inflammation and oxidative stress, which contribute to endothelial dysfunction and accelerate atherosclerotic processes, as well as osteogenic differentiation of vascular smooth muscle cells, leading to calcium deposition within the arterial wall. In addition, metabolic abnormalities commonly associated with lower eGDR, such as hyperglycemia, central obesity, and hypertension, may further exacerbate vascular injury and accelerate calcification (26, 27). Dysregulation of calcium–phosphate metabolism may also contribute to this process.

Consistent with these mechanisms, previous studies have shown that lower eGDR levels are associated with adverse cardiometabolic outcomes, including peripheral vascular disease and coronary artery disease (28, 29), and that insulin resistance is directly associated with increased AAC burden (10, 17). Despite these findings, evidence linking eGDR to structural vascular changes, particularly AAC, remains limited. AAC represents a late stage of atherosclerosis and reflects cumulative vascular damage. Our study extends previous research by demonstrating a consistent inverse association between eGDR and severe AAC across two independent cohorts. Taken together, these findings suggest that lower eGDR may promote vascular calcification through multiple biological pathways, whereas higher eGDR, reflecting better insulin sensitivity, may mitigate these pathological processes and reduce the risk of severe AAC.

Subgroup analyses provided additional insights into the consistency and potential heterogeneity of the association across different populations. Compared with previous studies, which primarily focused on overall associations, our analysis offers a more detailed evaluation of effect modification across multiple demographic and lifestyle factors, thereby extending the existing evidence. In the NHANES cohort, the association between higher eGDR and lower CVD risk appeared protective across age strata, whereas in the validation cohort the association was more pronounced among younger participants. This difference may reflect age-related variation in metabolic reserve capacity. Younger individuals may retain greater physiological flexibility in insulin sensitivity, such that early metabolic improvements confer larger benefits in preventing vascular damage and calcification (30, 31). In contrast, older individuals may have more advanced or irreversible vascular changes, potentially attenuating the impact of metabolic factors. We also observed that the association between eGDR and severe AAC was modified by education level, with stronger inverse associations among individuals with higher education. Differences in health literacy, access to healthcare resources, and long-term exposure to cardiometabolic risk factors may partly explain this pattern (32, 33). Notably, obesity status did not significantly modify the association in either cohort, suggesting that eGDR may capture metabolic risk beyond conventional anthropometric measures such as BMI. In lifestyle-related subgroups, stronger inverse associations were generally observed among never-smokers and moderate alcohol consumers, which is consistent with prior evidence (34, 35). Importantly, while the magnitude of associations varied across subgroups, the overall direction remained consistent, further supporting the robustness of our findings. These results provide additional evidence that the association between eGDR and severe AAC is not only stable but also exhibits population-specific variations, which may have implications for targeted risk stratification and personalized prevention strategies.

Current cardiovascular prevention strategies emphasize the early identification and management of metabolic risk factors, including obesity, hypertension, and dysglycemia, to reduce long-term vascular complications (36). Given that AAC is a strong predictor of cardiovascular morbidity and mortality, it represents an important target for early risk stratification. In this context, our findings suggest that eGDR may serve as a practical and accessible marker for identifying individuals at increased risk of severe AAC, particularly those without overt cardiovascular disease. Because eGDR is derived from routinely collected clinical parameters, including waist circumference, hypertension status, and HbA1c, it can be readily applied in both clinical and primary care settings. The incorporation of eGDR into routine clinical assessment may facilitate earlier detection of high-risk individuals and support more targeted preventive strategies. Individuals with lower eGDR levels may benefit from intensified lifestyle and metabolic interventions, such as weight management and glycemic control, which have been shown to improve vascular health and reduce cardiovascular risk (37–39). More studies and intervention trials intended at increasing or maintaining higher eGDR levels would be required to ascertain whether this exercise would lead to a reduction in AAC progression and cardiovascular benefit. However, whether improving eGDR translates into reduced progression of vascular calcification remains uncertain. Future prospective studies and interventional trials are warranted to determine whether targeting insulin resistance, as reflected by eGDR, can effectively mitigate AAC progression and improve cardiovascular outcomes.

This study has several important strengths. First, it extends previous research by incorporating an independent external validation cohort, which substantially enhances the robustness and generalizability of the findings. Second, by re-evaluating the dose–response relationship, our study provides new evidence supporting a stable and approximately linear association between eGDR and severe AAC. Third, we employed a transparent participant selection process with clearly defined inclusion and exclusion criteria, improving reproducibility and methodological rigor. Finally, comprehensive multivariable adjustment, dose–response analyses, and subgroup analyses demonstrating consistent associations across diverse populations further strengthen the reliability of our findings.

Several limitations should also be acknowledged. Due to the cross-sectional design, causal relationships cannot be established (40). It is necessary to conduct longitudinal studies to see whether the lower eGDR which precedes the development or progression of vascular calcification. Second, there may still be residual confounding even after adjusting for multiple demographic, socioeconomic, lifestyle and clinical covariates. Third, the investigators evaluated eGDR at a single time but did not consider its changes over time on insulin sensitivity, which may have weakened associations. It would be beneficial to conduct future prospective studies in various populations and ethnicities to validate our findings and determine whether an improvement in eGDR over time can mitigate vascular calcification.

In summary, our study provides robust evidence that lower eGDR levels are independently associated with a higher prevalence of severe AAC across two distinct populations. By integrating a nationally representative dataset with an independent external validation cohort, our findings not only confirm previous observations but also extend them by demonstrating a consistent and approximately linear association. These results highlight the potential of eGDR as a practical and accessible marker for identifying individuals at high risk of vascular calcification in both clinical and population settings.

## Conclusion

In conclusion, lower estimated glucose disposal rate is independently and linearly associated with severe AAC in both a nationally representative population and an external validation cohort. These findings suggest that eGDR may serve as a relatively simple marker for identifying individuals at high risk of vascular calcification.

## Data Availability

Publicly available datasets were analyzed in this study. This data can be found at: Centers for Disease Control and Prevention (CDC) website (https://www.cdc.gov/nchs/nhanes/).
