# Supplementary material for: Estimated glucose disposal rate and severe abdominal aortic calcification: evidence from a nationally representative study with external validation
Source: Front Nutr. 2026 Jun 19;13:1790028. doi: 10.3389/fnut.2026.1790028 (PMC13327944; doi:10.3389/fnut.2026.1790028)
Supplement: Supplementary file 1 [file Table_1.DOCX]

**Supplementary Material**

**Table S1.** Definition of variables involved in this study.

| Variables | Description in NHANES |
| --- | --- |
| Age | Divided into three groups: 20-65 years old, ＞65 years old |
| Sex | Male and Female |
| Race | Mexican American, Non-Hispanic Black, Non-Hispanic White, Other Race |
| Educational level | Below high school, High School, or above |
| Marital status | Yes: Married/Living with partner |
| PIR | Poor: <1.3; Not Poor:>=1.3 |
| CCI | <1, Participants who do not report whether they suffer from a certain disease as healthy |
| Obesity | Yes: BMI>=30 |
| Smoking | Smoking status was grouped into never smoker (defined as <100 cigarettes in a lifetime), current smoker (defined as ≥100 cigarettes in a lifetime), and former smoker (defined as ≥100 cigarettes and had quit smoking) |
| Drinking | heavy drinking (≥4 drinks/day for men, ≥3 drinks/day for women, or ≥5 days of drinking in a month),  moderate drinking (≥3 drinks/day for men, ≥2 drinks/day for women, or ≥2 days of drinking in a month),  mild drinking (≤2 drinks/day for men, ≤1 drink/day for women, and ≥12 drinks in a year),  and never-drinking (total number of drinks in a year <12, and dietary alcohol content of 0%) |

PIR, poverty income ratio; CCI, Charlson Comorbidity Index.

**Table S2.** Charlson Comorbidity Index Scores of various diseases included in this study.

| **Disease** | **Score** |
| --- | --- |
| Diabetes | 1 |
| Diabetic retinopathy | 2 |
| Kidney failure | 2 |
| Kidney stones | 2 |
| Heart failure | 1 |
| Stroke | 1 |
| Hepatopathy | 2 |
| Rheumatoid arthritis | 1 |
| Bladder cancer | 2 |
| Bone cancer | 2 |
| Brain cancer | 2 |
| Breast cancer | 2 |
| Cervical cancer | 2 |
| Colon cancer | 2 |
| Esophageal cancer | 2 |
| Gallbladder carcinoma | 2 |
| Kidney cancer | 2 |
| Tracheal carcinoma | 2 |
| Leukemia | 2 |
| Liver cancer | 2 |
| Lung cancer | 2 |
| Lymphomas | 2 |
| Melanoma | 2 |
| Oral cancer | 2 |
| Never cancer | 2 |
| Ovarian cancer | 2 |
| Pancreatic cancer | 2 |
| Prostatic cancer | 2 |
| Rectal cancer | 2 |
| Skin cancer(non-melanoma) | 2 |
| Other skin cancer | 2 |
| Soft tissue cancer | 2 |
| Stomach cancer | 2 |
| Testicular cancer | 2 |
| Thyroid cancer | 2 |
| Endometrial cancer | 2 |
| Other cancer | 2 |
